# Genetic variants in *ATM*, *H2AFX* and *MRE11* genes and susceptibility to breast cancer in the polish population

**DOI:** 10.1186/s12885-018-4360-3

**Published:** 2018-04-20

**Authors:** Marta Podralska, Iwona Ziółkowska-Suchanek, Magdalena Żurawek, Agnieszka Dzikiewicz-Krawczyk, Ryszard Słomski, Jerzy Nowak, Agnieszka Stembalska, Karolina Pesz, Maria Mosor

**Affiliations:** 1 Institute of Human Genetics, Polish Academy of Sciences, Poznan, Poland; 2 Department of Genetics, Wrocław Medical University, Wroclaw, Poland; 3 University of Life Sciences of Poznan, Poznan, Poland

**Keywords:** *ATM*, *H2AFX*, *MRE11*, DNA repair, Breast cancer

## Abstract

**Background:**

DNA damage repair is a complex process, which can trigger the development of cancer if disturbed. In this study, we hypothesize a role of variants in the *ATM*, *H2AFX* and *MRE11* genes in determining breast cancer (BC) susceptibility.

**Methods:**

We examined the whole sequence of the ATM kinase domain and estimated the frequency of founder mutations in the *ATM* gene (c.5932G > T, c.6095G > A, and c.7630-2A > C) and single nucleotide polymorphisms (SNPs) in *H2AFX* (rs643788, rs8551, rs7759, and rs2509049) and *MRE11* (rs1061956 and rs2155209) among 315 breast cancer patients and 515 controls. The analysis was performed using high-resolution melting for new variants and the polymerase chain reaction-restriction fragment length polymorphism (PCR-RFLP) method for recurrent *ATM* mutations. *H2AFX* and *MRE11* polymorphisms were analyzed using TaqMan assays. The cumulative genetic risk scores (CGRS) were calculated using unweighted and weighted approaches.

**Results:**

We identified four mutations (c.6067G > A, c.8314G > A, c.8187A > T, and c.6095G > A) in the *ATM* gene in three BC cases and two control subjects. We observed a statistically significant association of *H2AFX* variants with BC. Risk alleles (the G of rs7759 and the T of rs8551 and rs2509049) were observed more frequently in BC cases compared to the control group, with *P* values, odds ratios (OR) and 95% confidence intervals (CIs) of 0.0018, 1.47 (1.19 to 1.82); 0.018, 1.33 (1.09 to 1.64); and 0.024, 1.3 (1.06 to 1.59), respectively. Haplotype-based tests identified a significant association of the *H2AFX* CACT haplotype with BC (*P* <  0.0001, OR = 27.29, 95% CI 3.56 to 209.5). The risk of BC increased with the growing number of risk alleles. The OR (95% CI) for carriers of ≥ four risk alleles was 1.71 (1.11 to 2.62) for the CGRS.

**Conclusions:**

This study confirms that *H2AFX* variants are associated with an increased risk of BC. The above-reported sequence variants of *MRE11* genes may not constitute a risk factor of breast cancer in the Polish population. The contribution of mutations detected in the *ATM* gene to the development of breast cancer needs further detailed study.

## Background

Cell-cycle checkpoints and DNA damage repair prevent genetic instability and mutagenesis. In response to DNA double-strand breaks, a signaling cascade is initiated: first, the M/R/N complex, consisting of three proteins, MRE11, RAD50 and NBN, acts as a sensor for DNA damage. M/R/N proteins recruit the key signal transducer of DNA damage response: ataxia-telangiectasia mutated (ATM) kinase [1]. Activation of ATM causes cell cycle arrest. ATM phosphorylates several substrates, including histone H2AFX. The phosphorylated form of H2AFX, γ-H2AFX, modulates DNA repair mechanisms by reorganizing chromatin and preventing the separation of broken DNA ends.

Several genes involved in maintaining and monitoring genomic stability have emerged as breast cancer (BC) susceptibility genes. High-throughput methods have allowed identification of variants associated with breast cancer in more than 20 genes involved in DNA damage signaling and repair [2]. *BRCA1*, *BRCA2* and *CHEK2* are known breast cancer predisposition genes. Mutations in *BRCA1* or *BRCA2* have been detected in 20% of families with a history of breast cancer in Poland. Polish founder mutations (5382insC, C61G and 4153delA) are reported to be responsible for nearly 90% of *BRCA1* mutations [3, 4]. Furthermore, variants of *CHEK2* (1100delC, IVS2 + 1G/A, del5395bp, and I157T), *PALB2* (509_510delGA and 172_175delTTGT) and *RECQL* (c.1667_1667 + 3delAGTA) are also associated with breast cancer in the Polish population. Patients with *CHEK2* mutations have a greater-than-25% risk of breast cancer [5, 6]. The presence of *PALB2* mutations is associated with increased breast cancer risk (odds ratio [OR] = 4.4, 95% confidence interval [CI] 2.30 to 8.37; *P* <  0.0001) [7]. Moreover, a mutation in the *RECQL* gene is associated with a 5.5-fold increase in the risk of breast cancer in Poland [8]. In addition, individuals with certain rare genetic syndromes, such as Peutz-Jeghers (caused by *STK11* mutations, where the risk of BC is 45% by the age of 70) or Li-Fraumeni (caused by *TP53* mutations, with a BC relative risk of 6.4×), have an increased risk of breast cancer [9, 10].

Pathogenic mutations in *BRCA1* and *BRCA2* genes explain ~ 30% of the cases of families with a high risk of cancer and ~ 15% of breast cancer familial relative risk [11]. The genetic background of breast cancer is still unknown in some of cases. There are some indications of a potential contribution of other genes involved in the DNA damage response to breast cancer risk, including *NBS1*, *ATM*, *H2AFX*, *BRIP1*, *BARD1*, *RAD51C* and *RAD51D* [12].

We hypothesize that variants in the *ATM*, *H2AFX* and *MRE11* genes may modulate a predisposition to breast cancer.

## Methods

### Study population

We collected blood samples from 315 non-selected female patients diagnosed with breast cancer. A total of 515 anonymous blood samples were used as a control population. The control group consisted of individuals attending for a screening check-up in hospital or were healthy blood donors with no history of medical illness. Patients were eligible for present study if they revealed no mutations in *BRCA1*, *BRCA2* and *CHEK2* genes. The baseline characteristics of the patients are shown in Table 1. The mean age of patients was 53 years (range 26–76 years). Invasive ductal carcinoma was the most common subtype of cancer (*n* = 191, 60.6%). Most of the tumors were II and undetermined grade (*n* = 91, 28.9%, *n* = 75, 23.8%, respectively). ER/PgR status was available for majority of our BC patients. The study was conducted with the approval of the Central Ethical Committee of the Ministry of Health in Poland, in accordance with the tenets of the Declaration of Helsinki (Decision no. 949/16). All patients signed informed consent forms.

**Table 1:** Clinical characteristic of selected breast cancer patients

All BC patients	
Characteristic	Value
Mean age at diagnosis (yrs.)	53
Histological subtype of breast cancer No. (%)	
Ductale	191 (60.6)
Lobulare	35 (11.1)
Tubular carcinoma	15 (4.8)
Ductalolobular	11 (3.5)
Tubuloductale	6 (1.9)
Solidum	2 (0.6)
Mucinosum	2 (0.6)
Metaplasticum	2 (0.6)
Unknown	51(16.2)
Tumor grade No. (%)	
G1	31 (9.8)
G2	91 (28.9)
G3	55 (17.5)
Gx	75 (23.8)
Unknown grade	63 (20)
Family history of cancers No. (%)	
Positive	101 (32.1)
Negative	171 (54.3)
Unknown status	43 (13.7)
T stage at diagnosis No. (%)	
T1	43 (13.7)
T2	143 (45.4)
T3	37 (11.7)
T4	27 (8.6)
Tx	7 (2.2)
Unknown T stage	58 (18.4)
ERstatus No. (%)	
ER positive	152 (48.2)
ER negative	102 (32.4)
ER unknown	61 (19.4)
PgR status No. (%)	
PgR positive	154 (48.9)
PgR negative	101 (32.1)
PgR unknown	60 (19)

*BC* = breast cancer patients, *ER* - estrogen receptor, *PgR*- progesterone receptors

### Genotyping and mutation screening

Genomic DNA was extracted from whole blood samples using a PureGene DNA isolation kit in accordance with the manufacturer’s protocols (Gentra Systems).

The ATM mutations analysis was done using a combination of different methods. Polymerase chain reaction-restriction fragment length polymorphism (PCR-RFLP) was used to detect c.5932G > T, c.6095G > A and c.7630-2A > C mutations. PCR-RFLP analysis was performed using the restriction enzymes *MseI*, *BfaI* and *AluI*, respectively. The c.7630-2A > C mutation abolishes an *AluI* site. Digestion with *AluI* of the PCR product without the mutation gives four fragments (143, 70, 61 and 7 base pair (bp)), whereas the PCR product with mutation only three bands are observed (213, 61 and 7 bp). The c.5932G > T mutation creates an *MseI* restriction site. After digestion with *MseI*, a 232 bp PCR product produces three bands: 159, 40 and 33 in patients with the mutation, while c.6095G > A disrupts the *BfaI* site. After digestion with *BfaI*, a 234 bp PCR product without mutation shows two bands, 127 and 107 bp, and the PCR product of alleles with this mutation remains undigested. PCR products were digested with 10 U of restriction enzymes by overnight incubation at 37 °C. The restriction fragments were resolved on 3% agarose gel.

The sequence of the ATM kinase domain was analyzed using high-resolution melting (HRM). The primer sequences are listed in Supplement 1. The primers encompass a kinase domain sequence of *ATM* between codons 2712–2962. The PCR cycling and HRM analysis were done on CFX96 BioRad instruments. HRM was performed using a Type-it® HRM™ PCR kit (Qiagen, Crawley, UK) following the manufacturer’s instructions. The cycling protocol was as follows: 45 cycles of 95 °C for 10 s, 59 °C for 10 s, and 72 °C for 20 s; 1 cycle of 95 °C for 1 min; and a melt from 60 °C to 90 °C for all assays. For the melt, the temperature was increased at the rate of 0.2 °C/s. All reactions were carried out in duplicate.

Furthermore, selected variants of *H2AFX* and *MRE11* (rs7759, rs8551, rs643788, rs2509049, rs1061956 and rs2155209) were genotyped using TaqMan® SNP genotyping assays (Life Technologies, Carlsbad, California) and CFX96 BioRad instruments. Four SNPs, rs643788, rs8551, rs7759 and rs2509049, are located in the far promoter region of the *H2AFX* gene -1654C > T, -1420C > T, −1187A > G, and -417C > T, respectively. The PCR was performed with HOT FIREPol Probe qPCR Mix Plus (no ROX) in accordance with the manufacturer’s instructions (Solis Biodyne, Tartu, Estonia). The PCR thermal cycling was as follows: initial denaturation at 95 °C for 15 min. and next 40 cycles of 95 °C for 15 s and 60 °C for 60 s. As a quality control measure, negative controls and approximately 5% of the samples were genotyped in duplicate to check genotyping accuracy.

The genotypes of selected samples and newly detected *ATM* variants were confirmed by direct sequencing.

Nucleotide positions were determined according to the standard reference sequences for *ATM* NM_000051.3, whereby mutation numbering uses the ‘A’ of the ATG initiation codon as + 1. The reference sequence for *H2AFX* used NC_000011.10, and for *MRE11* NC_000011.9.

### Statistical analysis

All statistical analysis was undertaken using GraphPad Prism 5.0 software (GraphPad, La Jolla, CA, USA). The genotype frequencies of each SNP were tested for deviation from the Hardy-Weinberg equilibrium (HWE) amongst the controls. This was done by comparing the observed genotype frequencies with the expected frequencies using a Chi-squared test. The ORs and 95% CIs were calculated to assess BC risk. We considered *P* < 0.05 to be significant for all analyses. *P* values were corrected using Benjamini-Hochberg adjustment. Linkage disequilibrium (LD) measures (Lewontin’s D’ and the r^2^ coefficient) between SNPs were calculated using Haploview 4.2 software (Daly Lab, USA). Haplotype frequencies were compared among patients and controls (using the Chi-squared test). The statistical power analyses were determined using free available Power and Sample Size Calculator.

### Cumulative genetic risk score

SNPs showing significant association with BC were included in the cumulative genetic risk score (CGRS) analysis. Genotypes were coded as 0, 1 or 2, indicating the number of risk alleles in the genotype. Both unweighted (uwCGRS) and weighted (wCGRS) CGRS were calculated. In an unweighted approach, coded genotypes were counted to create a CGRS (therefore, the range of possible scores for three SNPs was 0 to 6). In a weighted approach, all the scores of the coded genotypes were multiplied by the log(OR) estimated for each risk allele in the current study. A weighted risk score is the sum of the multiplied results for each SNP and scaled by a factor of 3/∑w_i_, where w_i_ = log(OR) (the logarithm of the odds ratio) for the ith SNP and *i* = 3 [13]. The effect of unweighted and weighted CGRSs on BC was calculated using logistic regression analysis. A t-test was applied to compare the average and mode values of uwCGRS and wCGRS between the BC and control groups.

## Results

### *ATM* variants in BC patients

In the group studied, we found six changes (five in the BC patients and two in the control group) in the functional domain of the *ATM* gene (c.6067G > A, c.6095G > A (twice), c.8187A > T, c.8314G > A, c.6083A > G and c.8787-55C > T). In the BC cases, three were known mutations: c.6095G > A, c.8187A > T and c.6067G > A. The patient with the c.6067G > A mutation was also a carrier of the *NBN* Ile171Val mutation. Moreover, c.8787-55C > T was found in a homozygous state. We detected two mutations in the control group: c.6095G > A and c.8314G > A.Deleterious consequences of all detected changes were scored using the following tools: SIFT, PolyPhen2 and MutationTaster Phylop. All these algorithms estimate the pathogenic effects of SNPs on protein in different ways. SIFT calculates score based on multiple sequence alignments. PolyPhen2 predicts the possible effects using multiple sequence alignments, 3D protein structures and residue contact information from secondary structure. Mutation taster Phylop evaluates disease-causing potential of sequence alterations comprising many aspects: evolutionary conservation, splice-site changes, loss of protein features and changes that might affect the amount of mRNA. The pathogenic effects on ATM were confirmed if more than two analysis indicated damaging consequences. The details are presented in Table 2.

**Table 2:** The predicted effects of the *ATM* variants using SIFT, PolyPhen2 and Mutation taster Phylop algorithms

	SIFT	PolyPhen 2	Mutation taster Phylop
ATM			
c.6067G > A; G2023R	damaging; 0.03	benign,0.340	disease causing base on • amino acid sequence changed • heterozygous in tgp or exac • protein features (might be) affected
c.6095G > A; R2032K	tolerated; 0.1	possibly damaging with a score of 0.859	disease causing base on • amino acid sequence changed • known disease mutation at this position (hgmd cm990215) • known disease mutation at this position (hgmd cs961479) • known disease mutation: rs139770721 (pathogenic) • protein features (might be) affected • splice site changes
c.8187A > T; Q2729H	damaging; 0	probably damaging with a score of 1.000	disease causing base on • amino acid sequence changed • protein features (might be) affected • splice site changes
c.8314G > A; G2772R	damaging; 0.04	probably damaging with a score of 0.998	disease causing base on • amino acid sequence changed • protein features (might be) affected • splice site changes
c.6083A > G; Q2028R	damaging; 0.03	benign with a score of 0.232	polymorphism base on • amino acid sequence changed • protein features (might be) affected • splice site changes
c.8787-55C > T	intronic variant		polymorphism base on • homozygous in TGP or ExAC

SIFT algorithm ranges from 0 to 1. The amino acid substitution is predicted damaging is the score is ≤0.05, and tolerated if the score is > 0.05; PolyPhen 2 algorithm ranges from 0 to 1. possibly damaging and probably damaging (> 0.5) or benign (< 0.5); Mutation taster Phylop algorithm evaluates disease-causing potential of sequence alterations comprising evolutionary conservation, splice-site changes, loss of protein features and changes that might affect the amount of mRNA.

### *H2AFX* and *MRE11* genotype and allele distributions among patients and controls

The genotype and allele frequencies are summarized in Tables 3 and 4. The observed genotype frequencies of the six polymorphisms referred to above were all in agreement with the HWE in the control subjects (the *P* values for the *H2AFX* HWE were as follows: 0.32, 0.82, 0.08 and 0.72; for the *MRE11* HWE, the *P* values were: 0.84 and 0.59). For the *H2AFX* polymorphisms, the logistic regression analysis revealed that the rs7759 GG and rs8551 TT were significantly increased among breast cancer patients compared to the rs7759 AA and rs8551 CC genotypes, respectively (rs7759 GG versus AG: adjusted OR = 1.94, 95% CI 1.14 to 3.28; *P* = 0.042, after Benjamini-Hochberg correction; rs8551 CC versus TT, OR = 1.82, 95% CI 1.18 to 2.80; *P* = 0.034, after Benjamini-Hochberg correction). The significant association was also found between the *H2AFX* rs7759 polymorphism and cancer risk under the heterozygous co-dominant model (AA versus AG): OR = 1.73, 95% CI 1.28 to 2.33; *P* = 0.024, after Benjamini-Hochberg correction and under the dominant genetic model (AA versus AG + GG): OR = 1.76, 95% CI 1.32 to 2.34; *P* = 0.0016, after Benjamini-Hochberg correction. The G allele at rs7759 was significantly more prevalent in BC cases compared to the controls (OR = 1.47, 95% CI 1.19 to 1.82; *P* = 0.0018, after Benjamini-Hochberg correction). Otherwise, there were no differences at rs8551 under the dominant genetic model (CC versus CT + TT): OR = 1.37, 95% CI 1.021 to 1.83; *P* = 0.0357, or under the heterozygous co-dominant model (CC versus CT): OR = 1.26, 95% CI 0.93 to 1.71; *P* = 0.14. We observed that the frequency of the T allele of rs8551 was higher in BC patients than in the controls (OR = 1.33, 95% CI 1.09 to 1.64; *P* = 0.018, after Benjamini-Hochberg correction). There were no differences in the occurrences of the TC, CC and TC + CC genotypes at rs643788 and the CT, TT and CT + TT genotypes at rs2509049 between the BC cases and the controls. On the other hand, the T allele in rs2509049 was significantly higher in BC patients compared to controls: OR = 1.3, 95% CI 1.06 to 1.59; *P* = 0.024, after Benjamini-Hochberg correction. For the two *MRE11* variants, there were no statistically significant differences in genotype and allele frequencies between the BC patients and the controls at rs1061956 or rs2155209.

**Table 3:** Logistic regression analysis of the associations of the *H2AFX* and *MRE11* SNPs with BC

SNP	Genotypes No. (%)		*P* value	OR	95% CI
	C	BC			
H2AFX					
rs7759					
AA	263(51)	117 (37)		1^a^	
AG	217 (42.1)	167 (53.2)	**0.0003 (0.0024)** ^b^	1.73	1.28 to 2.33
GG	36 (6.9)	31 (9.8)	**0.013 (0.042)** ^b^	1.94	1.14 to 3.28
AG + GG	253 (49)	198 (63)	**0.0001 (0.0016)** ^b^	1.76	1.32 to 2.34
rs8551					
CC	220 (42.7)	111 (35.3)		1^a^	
CT	235 (45.7)	149 (47.2)	0.14	1.26	0.93 to 1.71
TT	60 (11.6)	55 (17.5)	**0.0063 (0.034)** ^b^	1.82	1.18 to 2.80
CT + TT	295 (57.3)	204 (64.7)	0.03	1.37	1.03 to 1.83
rs643788					
TT	228(44.4)	142 (45)		1^a^	
TC	216 (42)	118 (37.5)	0.40	0.88	0.65 to 1.19
CC	71 (13.6)	55 (17.6)	0.29	1.24	0.83 to 1.87
TC + CC	287 (55.7)	173 (55.1)	0.82	0.96	0.73 to 1.28
rs2509049					
CC	218 (42.3)	113 (35.9)		1^a^	
CT	237 (46)	147 (46.8)	0.25	1.19	0.88 to 1.62
TT	60 (11.7)	55 (17.3)	**0.009 (0.036)** ^b^	1.77	1.14 to 2.72
CT + TT	297 (58.6)	202 (64.1)	0.06	1.31	0.98 to 1.75
MRE11					
rs1061956					
AA	506 (98.2)	312 (99)		1^a^	
AG	9 (1.8)	3 (1)	0.35	0.54	0.15 to 2.01
rs2155209					
TT	247 (48)	149 (47.4)		1^a^	
TC	223 (43.2)	129 (41)	0.78	0.96	0.71 to 1.29
CC	45 (8.8)	37 (11.6)	0.21	1.36	0.84 to 2.20
CC + TC	268 (52)	166 (52.6)	0.85	1.03	0.78 to 1.36

^a^ = reference category; OR (95% CI) = odds ratio (95% confidence interval); ^b^ = result statistically significant after Benjamini-Hochberg correction; *C* = controls, *BC* = breast cancer patients

Bold data are statistically significant

**Table 4:** Allele frequency distribution and logistic regression analysis of the *H2AFX* and *MRE11* SNPs in BC

SNP/Alleles	No. of alleles (%)		*P* value	OR	95% CI
	C	BC			
rs7759					
A	743 (72)	401 (63.6)		1^a^	
G	287 (28)	229 (36.4)	**0.0003 (0.0018)** ^b^	1.47	1.19 to 1.82
rs8551					
C	675 (65.6)	371 (58.9)		1^a^	
T	355 (34.4)	259 (41.1)	**0.006 (0.018)** ^b^	1.33	1.09 to 1.64
rs643788					
T	672 (65.2)	379 (60)		1^a^	
C	358 (34.8)	251(39.8)	0.16	1.16	0.94 to 1.43
rs2509049					
C	673 (65.3)	373 (59.3)		1^a^	
T	357 (34.6)	257 (42.2)	**0.012 (0.024)** ^b^	1.3	1.06 to 1.59
rs1061956					
A	1021(99.1)	627 (99.5)		1^a^	
G	9 (0.9)	3 (0.5)	0.35	0.54	0.15 to 2.01
rs2155209					
T	717 (69.6)	427 (67.8)		1^a^	
C	313 (30.4)	203 (32.2)	0.43	1.09	0.88 to 1.35

^a^ = reference category; OR (95% CI) = odds ratio (95% confidence interval); ^b^ = result statistically significant after Benjamini-Hochberg correction; *C* = controls, *BC* = breast cancer patients

Bold data are statistically significant

### Frequency of *H2AFX* promoter haplotypes and risk of breast cancer

**Fig. 1:**
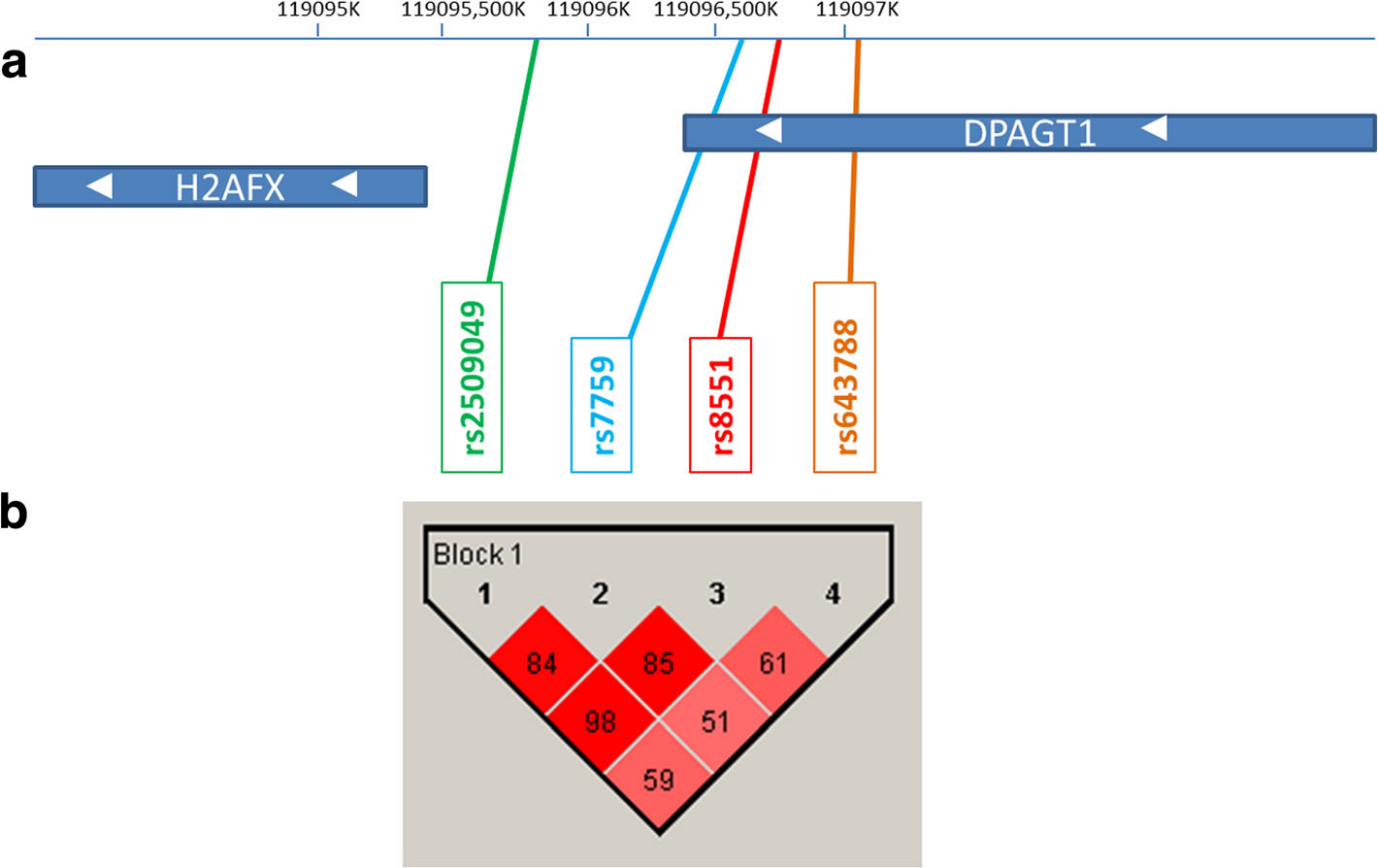
Pairwise linkage disequilibrium (LD) map between four SNPs of the *H2AFX* gene (Haploview 4.1). **a** Graphic overview of polymorphisms identified in relation to the *H2AFX* gene. **b** The colors represent the relative D’/LOD scores and the correlation coefficients (r^2^) are presented as values

To determine the combined effects of the four promoter *H2AFX* SNPs, we generated haplotypes based on the observed genotypes (Fig. 1). For *H2AFX* SNPs, the construction of haplotypes revealed the presence of seven haplotypes in BC patients and eight haplotypes in the control group. In both groups, the most frequent *H2AFX* haplotype was CACC, without any risk variant allele (54.4% in BC patients and 61.4% in the control group). We also observed haplotypes that only presented in particular groups: TGTC in BC cases and TGCT and CATC in the control group. This observation could be related to the small size of the study group. Haplotypes with a frequency lower than 1% were not considered for further analysis. Haplotypes are presented in Table 5. When the CACC haplotype was used as the reference, CACT haplotype was associated with an increased risk of breast cancer. The difference in the frequency distribution of haplotype between the BC cases and controls was statistically significant (*P* < 0.0001 for CACT: OR = 27.29, 95% CI 3.56 to 209.5).

**Table 5:** Haplotype frequencies detected in studied groups

Haplotypes	BC (%)	C (%)	*P* value	OR	95% CI
CACC	54.4	61.4		1^a^	
TGTT	31.5	27.8	0.1356	1.28	0.93 to 1.77
CACT	4.8	0.2	**< 0.0001 (0.0003)** ^b^	27.29	3.56 to 209.5
TATT	3.3	3.8	0.9492	1.026	0.4658 to 2.260

^a^ = reference category; OR (95% CI) = odds ratio (95% confidence interval); ^b^ = result statistically significant after Benjamini-Hochberg correction; *C* = controls, *BC* = breast cancer patients

Bold data are statistically significant

### Cumulative genetic risk score

**Fig. 2:**
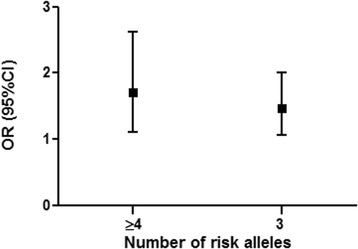
Cumulative genetic risk score analysis of *H2AFX* variants. The effect of weighted CGRS on BC was calculated using logistic regression analysis. The ORs (the black squares) with 95% confidence intervals (the black bars) for the number of risk alleles are from the weighted analysis

All *H2AFX* variants that showed a significant association with BC were included in the CGRS analysis. The risk alleles were defined as G for rs7759 and T for rs8551 and rs2509049. The average (± standard deviation [SD]) of the cumulative risk scores among the two groups studied were similar (for BC 2.37 ± 2.06 and for the control group 2.01 ± 1.88), although mode values were different for the BC and control groups, at 3 and 0 respectively. Individuals were stratified into three groups according to the number of risk alleles: carrying ≤ two (reference group), three, and ≥ four alleles. The risk of BC increased with the number of alleles and was statistically significant for three alleles (*P* = 0.019) and ≥ four alleles (*P* = 0.014) compared to ≤ two risk alleles. ORs (95% CI) for carriers of three and ≥ four risk alleles were 1.46 (1.06 to 2.01) and 1.71 (1.11 to 2.62), respectively (Fig. 2). The ORs calculated in unweighted and weighted CGRS analysis were similar, which is probably connected to the low number of risk alleles and small sample size. We also compared a clinical data between patients with high cumulative genetic risk score (≥4 risk alleles) and all BC patients. The detailed clinical parameters of BC patients with high cumulative genetic risk score (≥4 risk alleles) are shown in Table 6.

**Table 6:** Clinical characteristics of BC patients with high cumulative genetic risk score (≥4 risk alleles)

Characteristics	BC patients	
	with ≥4 risk alleles	with < 4 risk alleles
Mean age at diagnosis (yrs.)		
	53	54
Histological subtype of breast cancer No. (%)		
Ductale	55 (77.5)	136 (55.7)
Lobulare	6 (8.5)	29 (11.9)
Tubular carcinoma	2 (2.8)	13 (5.3)
Ductalolobular	–	11 (4.5)
Tubuloductale	6 (8.5)	–
Solidum	1 (1.4)	1(0.4)
Mucinosum	–	2 (0.8)
Metaplasticum	1 (1.4)	1(0.4)
Unknown	–	51(20.9)
Tumor grade No. (%)		
G1	–	31 (12.7)
G2	33 (46.5)	58 (23.8)
G3	13 (18.3)	42 (17.2)
Gx	25 (35.2)	50 (20.5)
Unknown grade	–	63 (25.8)
Family history of cancers No. (%)		
Positive	18 (25.4)	83 (34)
Negative	53 (74.6)	118 (48.4)
Unknown status	–	43 (17.6)
T stage at diagnosis No. (%)		
T1	16 (22.5)	27 (11.1)
T2	36 (50.7)	107 (43.9)
T3	10 (14.1)	27 (11.1)
T4	9 (12.7)	18 (7.4)
Tx	–	7 (2.9)
Unknown T stage	–	58 (23.6)
ERstatus No. (%)		
ER positive	25 (35.2)	127 (52)
ER negative	42 (59.2)	60 (24.6)
ER unknown	4 (5.6)	57 (23.4)
PgR status No. (%)		
PgR positive	30 (42.3)	124 (50.8)
PgR negative	39 (54.9)	62 (25.4)
PgR unknown	2 (2.8)	58 (23.8)

*BC* = breast cancer patients, *ER* = estrogen receptor, *PgR* = progesterone receptors

### Statistical power analysis

The post-hoc analysis revealed that the statistical power of our study for analyses of the differences in distribution of alleles at loci rs7759, rs8551 and rs2509049 (OR: 1.3 to 1.47 for the three *H2AFX* SNPs with a frequency of 0.28 to 0.35) between BC patients and controls ranged between 71 and 94%. This means that the study had enough power to detect an association of the *H2AFX* gene in BC group, in the case-control analysis.

## Discussion

Our previous studies focused on the hypothesis that M/R/N gene polymorphisms are associated with the risk of different cancers. We showed that the germline p.Ile171Val mutation in *NBN*, one of the M/R/N genes, may be considered a risk factor in the development of solid malignant tumors, including breast cancer, larynx and colorectal cancer or acute lymphoblastic leukemia (ALL) [14–17]. Heterozygous carriers of the *NBN* c.657del5 mutation have an increased risk of malignant tumor development, especially of breast, prostate, colon and rectal cancers [18]. We also demonstrated that *RAD50* gene mutations are not a risk factor of familial and sporadic breast cancer in the Polish population [19].

In this case-control study, we investigated the relationships between other variants in *ATM*, *H2AFX* and *MRE11* genes and risk of breast cancer.

It has been shown that heterozygous *ATM* mutations cause increased risk of malignancy. Female relatives of ataxia-telangiectasia cases have increased risk of breast cancer [20, 21]. Moreover, numerous epidemiological studies have indicated the contribution of *ATM* variants to breast cancer [22–26]. A few recurring mutations in the *ATM* gene have been detected in Polish ataxia-telangiectasia patients. Three of the mutations, c.6095G > A, c.7630-2A > C and c.5932G > T, were the most frequent [27, 28]. A mutation at position 5932 creates a stop codon and changes a GAA codon, specifying glutamine, into a UAA. A second mutation at position 6095 is the substitution of the last nucleotide of exon 43 and changes guanine to adenine. This mutation results in the deletion of exon 43, caused by defective splicing. The last mutation alters the splice-acceptor site at − 2 from exon 54 and results in a deletion of this exon beginning at codon 2544. Therefore, in this study, we investigated the frequency and spectrum variants of the kinase domain in the *ATM* gene in a series of women with breast cancer.

There are a few studies regarding associations between breast cancer development and the *ATM* gene mutation in the Polish population. Bogdanova et al. showed that the c.5932G > T mutation is a predisposing breast cancer susceptibility variant in populations in Belarus, Russia, Ukraine and Poland [29]. In another study, two protein-truncating mutations in the *ATM* gene were found in two Polish probands with breast cancer without founder mutations in *BRCA1*, *CHEK2* or *NBS1*. In that study, both patients with *ATM* mutations also had another truncating mutation, in the *PALB2* and *XRCC2* genes, respectively [30].

In the coding sequence of the *ATM* kinase domain in our study, we detected five mutations in the 830 samples in both the BC and control groups. One of the mutations, which presented in two BC patients, is the founder mutation (c.6095G > A) observed in Polish ataxia-telangiectasia patients. The rest of the detected variants were single nucleotide changes: c.6067G > A, p.Gly2023Arg; c.8314G > A, p.Gly2772Arg; c.8187A > T, p.Gln2729His; c.8787-55C > T and c.6083A > G; Q2028R. Using SIFT and PolyPhen and Mutation taster Phylop algorithms to predict the possible impact of the amino acid changes on ATM function, we confirmed that three of the missense variants (c.6067G > A, c.8187A > T, c.8314G > A) were classified as probably being damaging mutations/ disease causing. All these algorithms estimate a functional effect of SNPs in different ways. Accordingly, the pathogenic effects of *ATM* gene variants were confirmed if more than two analyses indicated demanding consequences.

However, one functional study indicated that c.8314G > A, p.Gly2772Arg is only a missense variant, which does not interfere with ATM kinase activity and radiosensitivity [31]. However, we cannot exclude the possibility that this mutation has an impact on the interaction between ATM and other proteins. Mutation c.6067G > A was observed in a patient from Brazil with sporadic breast cancer. In that case, the tumor was diagnosed at the age of 45 and was defined as clinical stage II [32]. In our case tumor was diagnosed at the age of 49 and the pathologic stage of tumor was defined as T2N1M0. The c.8187A > T variant was identified in one case of familial prostate cancer [33]. Moreover, our patient with c.6067G > A was also a carrier of the *NBN* p.Ile171Val mutation. It is difficult to conclude which changes are pathogenic because the p.Ile171Val variant has been connected with ALL, breast, larynx and colorectal cancer, and multiple primary tumors of the head and neck [34–37]. On the other hand, Dzikiewicz-Krawczyk et al. indicated that the heterozygous p.Ile171Val mutation does not significantly impair nibrin function and, therefore, p.Ile171Val does not play a crucial role in tumorigenesis [38].

In the above-mentioned results and in data previously presented by Cybulski et al., it was observed that some of the BC patients with detected *ATM* variants also had other changes in different genes involved in DNA damage repair [30]. This evidence suggests that, in some BC cases, the development of breast cancer can be linked with the accumulation of variants in DNA damage repair genes.

In addition, we found two polymorphic variants: c.6083A > G, Gln2028Arg and the intronic variant, c.8787-55C > T, which was found in a homozygous state. These two variants do not play a role in the development of breast cancer.

In the second part of our case-control study, six potentially functional SNPs were genotyped in two other genes, *H2AFX* and *MRE11*, connected with the DNA damage response signaling cascade. We selected SNPs in *H2AFX* and *MRE11* genes based on observations from previous reports [39–42]. Four SNPs, rs643788, rs8551, rs7759 and rs2509049, are located in the far promoter region of the *H2AFX* gene -1654C > T, -1420C > T, −1187A > G, and -417C > T, respectively. Two of SNPs, rs8551 and rs7759, are also located in the 3′UTR of other gene, *DPAGT1*. While, the rs643788 causes an amino acid change in DPAGT1 protein. This substitution converts isoleucine into valine (I393V). The I393V variant was predicted as tolerated by SIFT and benign by PolyPhen2. *DPAGT1* gene encodes an enzyme that catalyzes the first step in the dolichol-linked oligosaccharide pathway for glycoprotein biosynthesis. However, we did not find any evidences that I393V variant has pathogenic effect on DPAGT1 protein or is associated with an increased risk of cancer.

The *MRE11* variants, rs1061956 (*442A > G) and rs2155209 (*2501A > G), are located in non-coding DNA sequences: the three prime untranslated region (3’UTR) of the gene. A functional study of polymorphisms in the *H2AFX* distal promoter showed a possible regulatory impact of two SNPs. Studies, based on gel shift assays, revealed that the rs643788 C allele disrupts a consensus sequence for a Yin Yang 1 transcription factor binding site. Moreover, the probe with rs2509049 C allele binds more strongly to an undefined protein complex than the rs2509049 T allele. On the other hand, it has been shown no differential binding by gel shift assay for rs8551 and rs7759 probes. It is not excluded that these SNPs may have an impact on binding only under specific conditions [43]. A few studies have indicated that SNPs in the promoter region of *H2AFX* are associated with cancer risk. Lu et al. found significant associations between minor variant genotypes of four SNPs (rs643788, rs8551, rs7759 and rs7350) and haplotypes with minor alleles in the promoter region of *H2AFX* and risk of breast cancer. Age at onset of breast cancer significantly decreased as the number of variant alleles in the *H2AFX* promoter region increased [44]. Furthermore, Novik et al. indicated the protective effect of the rs2509049 TT genotype in non-Hodgkin lymphoma [41].

Our findings suggest that there is a potential link between an increased risk of breast cancer and two *H2AFX* SNPs: rs8551 and rs7759. Likewise, comparing the allele frequency of rs7759, rs8551 and rs2509049 SNPs, we observed a statistically significant higher prevalence of the minor alleles in BC cases in comparison with the control group. However, the haplotype analysis of all the *H2AFX* polymorphisms studied showed no association of haplotypes with minor/major alleles with increased risk of breast cancer. We only observed significant differences in the distributions of haplotype consisting of CACT alleles between the BC cases and controls.

We also identified a cumulative effect of three SNPs in the *H2AFX* promoter locus. The risk of breast cancer escalated with an increased number of risk alleles. The comparison of clinical data between two groups, BC patients with high cumulative genetic risk score (≥4 risk alleles) and BC patients with < 4 risk alleles, showed that high CGRS is not correlated with age of diagnosis (53 vs 54 yrs.), T stage and histological subtype of breast cancer. We observed differences in tumor grade among two groups. In patients with high cumulative genetic risk score, grades of tumor were shifted towards moderate and poor differentiation (% of tumor grades G1 vs G2 + G3, 0% vs 64.8% in patients with high cumulative genetic risk score; 12.7% vs 41% in BC patients with < 4 risk alleles). We found increased numbers of patients with high cumulative genetic risk score with negative ER status (59.2% vs 24.6%) and PgR status (54.9% vs 25.4%), in comparison to all BC cases.

In this paper, two other SNPs from a subsequent gene involved in the DNA repair process, the *MRE11* gene, were investigated. Choudhury et al. demonstrated the *MRE11* 3’UTR SNP to be associated with bladder cancer risk. However, the authors noticed a marginal increase in risk of bladder cancer for rs2155209 (OR = 1.54, 95% CI 1.13 to 2.08; *P* = 0.01) in individuals homozygous for the C allele compared to those carrying the common TT or TC genotype [42]. The carrier state of at least one rare 3’UTR variant of *MRE11* was significantly associated with worse cancer-specific survival among patients with muscle-invasive bladder cancer [45]. In this report, there is a lack of association of *MRE11* polymorphisms with breast cancer patients from Poland. Neither the rs2155209 nor the rs1061956 SNP showed statistically significant differences in the frequencies of genotypes.

## Conclusion

The current data suggest that *H2AFX* variants are significantly associated with BC. The risk of BC increased with the number of the risk alleles (G of rs7759, T of rs8551 and T of rs2509049) carried. The above-reported sequence variants of the *MRE11* gene may not constitute a risk factor of breast cancer in the Polish population. The contribution of mutations detected in the *ATM* gene to the development of breast cancer needs further detailed study.

## Abbreviations

ATM: Ataxia-telangiectasia mutated
CGRS: Cumulative genetic risk score
H2AFX: H2A histone family, member X
HRM: High-resolution melting
HWE: Hardy-Weinberg equilibrium
LD: Linkage disequilibrium
M/R/N: Mre11/Rad50/NBN
MRE11: Homolog, double-strand break repair nuclease
PCR-RFLP: Polymerase chain reaction-restriction fragment length polymorphism
SNP: Single nucleotide polymorphism
